# Three discipline collaborative radiation therapy (3DCRT) special debate: Peer review in radiation oncology is more effective today than 20 years ago

**DOI:** 10.1002/acm2.13103

**Published:** 2020-11-24

**Authors:** Anis Ahmad, Lakshmi Santanam, Abhishek A. Solanki, Laura Padilla, Erina Vlashi, Patrizia Guerrieri, Michael M. Dominello, Jay Burmeister, Michael C. Joiner

**Affiliations:** ^1^ Department of Radiation Oncology University of Miami Miami FL USA; ^2^ Department of Radiation Oncology Memorial Sloan Kettering Cancer Center New York NY USA; ^3^ Department of Radiation Oncology Loyola University Maywood IL USA; ^4^ Department of Radiation Oncology Virginia Commonwealth University Richmond VA USA; ^5^ Department of Radiation Oncology University of California Los Angeles CA USA; ^6^ Department of Radiation Oncology Allegheny Health Network Pittsburgh PA USA; ^7^ Department of Oncology Wayne State University School of Medicine Detroit MI USA; ^8^ Gershenson Radiation Oncology Center Barbara Ann Karmanos Cancer Institute Detroit MI USA

## 1 THREE DISCIPLINE COLLABORATIVE RADIATION THERAPY (3DCRT) DEBATE SERIES

Radiation Oncology is a highly multidisciplinary medical specialty, drawing significantly from three scientific disciplines — medicine, physics, and biology. As a result, discussion of controversies or changes in practice within radiation oncology must involve input from all three disciplines. For this reason, significant effort has been expended recently to foster collaborative multidisciplinary research in radiation oncology, with substantial demonstrated benefit.^1^, ^2^, ^3^ In light of these results, we endeavor here to adopt this “team‐science” approach to the traditional debates featured in this journal. This article is part of a series of special debates entitled “Three Discipline Collaborative Radiation Therapy (3DCRT)” in which each debate team includes a radiation oncologist, medical physicist, and radiobiologist. We hope that this format will not only be engaging for the readership but will also foster further collaboration in the science and clinical practice of radiation oncology.

## 2 INTRODUCTION

Technologic evolution in everything from treatment planning to treatment delivery, patient immobilization to on‐board imaging, complex simulation techniques to modified fractionation regimens, MR Linac to PET Linac, have changed our field exponentially in the past two decades. With these new technologies and abilities, a given radiotherapy patient’s treatment plan may be significantly more individualized and complex than it might have been 20 yr ago. The question we face today in this debate is whether peer review in radiation oncology, an accepted critical component of high‐quality and safe delivery of care, is more effective today than it was 20 yr ago. In the face of these significant changes, variability in technique, improvements, and innovations, have we maintained appropriate focus? During peer review do we still ask the right questions? Have we EVER asked the right questions and do we know what those right questions are? How much time is enough time reviewing a patient case? Do we spend more time now than we did 20 yr ago? Do we adequately focus on clinical factors: contours, fractionation, type of delivery, treatment time, patient limitations? How do we allocate our time in peer review? What components are highest yield or at highest risk for error and how have these trends changed for the better or worse in the past two decades? Have we learned any lessons in the past 20 yr when it comes to peer review and if so are they even relevant given the rapid changes in technology that we see year after year within our field? We all strive to deliver safe and effective radiotherapy. Has our peer review process kept pace with the ever‐changing technology or are our intentions overwhelmed by and lost on the complexity of a patient case in the year 2020? Let us debate!

Arguing for the proposition will be Drs Lakshmi Santanam, Abhishek Solanki, and Anis Ahmad. Dr Santanam is an attending medical physicist at Memorial Sloan Kettering Cancer Center. Her primary interests include motion management and patient safety. She currently serves as Chair for the AAPM Working Group on RO‐ILS and Vice‐Chair of the Task Group on the Management of Respiratory Motion in Radiation Oncology. Dr Solanki is Associate Professor, Quality Medical Director, Director of Clinical Research, and Chief of Genitourinary Radiotherapy at Loyola University. He joined the faculty of Stritch School of Medicine in 2014 after completing medical school and residency in radiation oncology at the University of Chicago. His clinical practice focuses primarily on genitourinary malignancies and he led the development of a prostate high dose rate (HDR) brachytherapy program at Loyola. Dr Solanki has a particular interest in quality and safety in radiation oncology, leading a multidisciplinary team to develop a prospective peer review program in the Loyola network, and is a member of the Veterans Affairs Radiation Oncology Quality Surveillance program. Dr Ahmad received his MPhil and PhD from Aligarh Muslim University, India followed by a postdoctoral fellowship at the Medical University of South Carolina. He has authored more than 30 peer‐reviewed scientific articles, and has been cited over 1200 times. He serves as Associate Editor for the Open Access Journal of Cancer & Oncology and review editor for Frontiers in Neurodegeneration. Dr Ahmad is now an Assistant Scientist with Sylvester Comprehensive Cancer Center at the University of Miami and his primary research focuses are radiation response of tumor and normal tissue to low and clinically relevant doses of radiation.

Arguing against the proposition will be Drs Laura Padilla, Erina Vlashi, and Patrizia Guerrieri. Dr Padilla is a medical physicist in the Department of Radiation Oncology at Virginia Commonwealth University. She has an Assistant Professor appointment and is the Associate Program Director of the Medical Physics graduate program. Her research focuses on uses of surface imaging in radiation oncology, workflow and process improvements, and new educational strategies in medical physics. Dr Vlashi received her PhD in Chemistry from Purdue University, followed by postdoctoral training in cancer stem cell biology in the Department of Radiation Oncology at UCLA, where she is now an Associate Professor. Dr Vlashi’s current research interests include investigating the effect of radiation on cell metabolism to identify targetable vulnerabilities that can be exploited for improving the clinical benefits of radiation therapy. Dr Guerrieri is Assistant Professor with the Department of Radiation Oncology of Allegheny Health Network, Pittsburgh. She earned her medical degree from the Universita’ Cattolica del Sacro Cuore, Rome, Italy and her Master of Science in Radiation Sciences at Hahnemann University, Philadelphia. As Coordinator of the Italian Group of Brachytherapy she was on the committee for the compilation of the Italian Association of Radiation Oncology guidelines on breast cancer. She served as president of the organizing committee and scientific director of the Post‐Graduate Teaching Course in Brachytherapy, Palermo 2006, and as scientific director and organizer of the National Interactive Course in Brachytherapy for Physicists and Radiation Oncologists, 2011.

## 3 OPENING STATEMENTS

### 3.A Lakshmi Santanam, PhD; Abhishek Solanki, MD; Anis Ahmad, PhD (FOR)

Peer review is a critical component of a radiation oncology quality management program.^4^, ^5^ A keyword search in Google for “Peer review in Radiation Oncology” now yields close to 31 400 results, compared to 6240 results from 1980 to 2000, which highlights the importance this topic has gained in the last 20 yr. With advances in automation, technology, remote review, and cloud computing, engaging multidisciplinary teams via teleconference to review patient contours, radiation treatment plans, and weekly chart rounds is more easily achievable now than in the past.

Peer review is more efficient now than it was 20 yr ago when it comes to planning quality,^6^ reducing variation in practice,^7^ identifying cancer sites with a high proportion of changes,^6^ developing or improving treatment planning policies,^7^ and promoting multidisciplinary communication and engagement.^8^

Although historically there were limited quantitative and qualitative data regarding the impact of peer review, during the past 10 yr there have been numerous studies describing the impact of multidisciplinary chart rounds on radiation treatment plans. A systematic review of 11 491 patient cases in 11 studies demonstrated that 10.8% of radiation treatment plans required modification as a result of peer review, with the top 3 causes being target volume change (45%), dose prescription or written directive (24%), and nontarget volume delineation or normal tissue sparing (7.5%).^9^

Many institutions have shifted to peer review earlier in the radiation therapy workflow because of a better understanding of the most common errors identified during peer review. Historically, most institutions have used a weekly “chart rounds” approach, in which patients undergo simulation, treatment planning, and begin treatment. Typically thereafter, the target contours, normal structure contours, and treatment plans, are reviewed during the first week of treatment.^10^ However, many institutions have evolved their peer review program to conduct peer review before the start of radiotherapy and as early as possible in the treatment planning process.^11^, ^12^, ^13^

There are several benefits to this newer approach to peer review:

1. Peer review and implementation of the changes earlier in the treatment planning process (i.e., before dosimetric planning) limits the issues that accompany having to “replan” a patient (i.e., strain on staff and resources and the potential for errors due to repeating work).
2. Peer review before the start of radiation therapy eliminates any suboptimal radiation therapy delivery. In contrast, peer review after the start of radiation therapy requires replanning and ultimately, any issues with the radiation plan identified during peer review for the index case can only be mitigated. Peer review before the start of radiation therapy is particularly important as the use of hypofractionated regimens has increased in radiation oncology. An example of this is the experience of the University of Michigan.^14^ Investigators developed a preplanning SBRT round and found that among 513 SBRT treatment courses, 22% had a change made before planning — thereby preventing replanning of these complicated plans and potentially preventing harm to patients. Preplanning SBRT round highlights how the shift to earlier peer review can prevent errors that could be clinically significant.
3. In addition to identifying errors in hypofractionated courses, where the effect of these errors could be amplified, studies suggest that peer review can lead to increased utilization of hypofractionated regimens. For example, a series from Banner MD Anderson Cancer Center in Arizona demonstrated that prospective peer review of palliative bone metastasis radiation courses led to increased utilization of 1–5 fraction regimens and decreased use of 10+ fraction regimens.^15^ Similarly, a study from the University of Kansas found that peer review led to increased use of hypofractionated regimens for early‐stage breast cancer at both the academic center as well as community‐based affiliates.^16^ As we move toward increased use of hypofractionation, peer review acts as a powerful vehicle to facilitate change.
4. A review of all curative radiotherapy treatment plans at 14 radiation oncology centers in Ontario revealed that peer review before the start of radiation was more likely to identify changes that were incorporated into the plan compared to peer review after the start of RT.^17^
5. Peer review before the start of radiation therapy allows for changes to be made in the overall multidisciplinary plan and incorporation of further diagnostic workup. The classic chart rounds review after the start of radiation therapy may identify inappropriate incorporation or omission of multidisciplinary treatments such as concurrent chemotherapy or surgery, or the exclusion of critical workup studies that may help with clinical risk stratification or even radiation treatment planning.
6. There is a greater understanding that peer review may need to be molded to specific disease entities. Studies have suggested that head and neck cancer patients have the highest frequency of changes due to the physician contours, and therefore centers increasingly put emphasis on early contour review for this patient population.^18^

From the physics perspective, pretreatment physics plan checks have been identified as one of the most effective individual quality control checks.^19^ Automating prescriptions, contour checks for normal contours, plan quality checks, transfer to EMR checks, and associating checklists has made most of these peer review interventions easier from the physicist and therapist perspectives.^20^

New auto‐segmentation tools to aid physicians in identifying targets, tools to determine if margins were done accurately, auto‐propagation of contours in 4D datasets, and other tools are becoming available in commercial systems. Although these tools exist as a guide for physicians, ultimately peer review by colleagues is essential and is being practiced by the majority of clinicians.

Another way in which peer review has improved over the last two decades is through the engagement of professional organizations to maximize peer review. ASTRO recently created a Peer Review website where physicians who might be looking for an expert consultant for advice regarding challenging cases or who do not have colleagues available in their practice for peer review can be connected to others. This initiative allows for peer review in settings where it may not have previously been possible and demonstrates the commitment to peer review among the radiation oncology community.

Identifying what needs to be peer reviewed, timelines, available resources, etc., are all critical factors that need to be determined for effective and efficient implementation. Despite the challenging nature of peer review implementation, it is more feasible now than ever before within the standard operations of every radiation oncology department.^21^

### 3.B Laura Padilla, PhD; Erina Vlashi, PhD; Patrizia Guerrieri, MD (AGAINST)

The indisputable importance of peer review for the quality and safety of healthcare delivery is emphasized in many publications.^22^, ^23^, ^24^, ^25^ The complexity of the radiation delivery tools and the biological response to radiation makes peer review particularly important in radiotherapy.^24^, ^25^

By the late 1980s, national standards developed by experts had established the key components of good quality control and assurance in radiation oncology.^23^ However, the need for structured peer review did not become evident until the mid‐1990s when Levitt & Khan, reviewing various clinical trials, showed that the weakest links in quality control and treatment outcomes were related to human factors.^25^ A few years later, two seminal publications laid the groundwork for modern peer review. The first, “To err is human: building a safer health system,”^24^ laid the foundation for a culture of safety in the healthcare system, and the second outlined the key elements of good peer review in radiation oncology based on the experience of the Regional Cancer Center in Kingston, Ontario.^22^

Today, it seems reasonable to think that the experience of conducting structured peer review for nearly three decades, would make modern peer review in radiation oncology more effective than 20 yr ago, especially when one factors in the sophisticated software available for analysis, retrieval, and display of patient information. Here we posit, however, that although modern peer review is certainly aided by advanced technology, its evolution has been outpaced by an exponential escalation in treatment complexity and the ever‐growing challenge of how to meaningfully design and integrate this activity into the clinical workflow. Below, we outline the factors we believe hamper the widespread implementation of effective peer review in today’s radiotherapy, thus challenging the proposition that modern peer review is more effective today than it was 20 yr ago.

*Complex treatment approaches have outgrown modern peer review*. As emphasized in “Safety is No Accident,”^26^ “as the field advances, traditional approaches, processes and workflows should be continually challenged and reassessed” and peer review is no different. The relative simplicity of radiotherapy treatment planning 20 yr ago accommodated for “effective peer review” by discussing every aspect of the treatment plan prior to the beginning of treatment (prospective review), including treatment indications, prescription, and port and verification films. In the early 2000s, most treatments used a standard schedule of 2 Gy/fraction, patient retreatments were less common, and the debate about different fractionation schedules was only beginning. Additionally, 3D Conformal Radiotherapy was considered a “complex treatment” and plans were not as sensitive to contouring inaccuracies.^23^, ^26^ This is no longer true today. Inaccurate contouring has been identified as one of the highest risk failure factors in a recent task group report for physics plan and chart review.^27^ Despite this, physicians are more likely to review prescription and overall treatment strategy during peer review than contours.^10^ When contours are reviewed, issues with contouring account for over half of the major changes requested during peer review.^21^, ^28^ This indicates a potential mismatch between the focus of some current peer review practices and the clinically impactful factors in modern radiotherapy. Peer review practices that do not prioritize, at the very least, target contour review are ineffective for modern radiotherapy techniques.

Contour sensitivity of modern plans is not the only added layer of complexity. Other factors, like image fusion for structure delineation and dose composite estimates, and the combination of treatments with wildly diverse fractionation schemes and of different modalities, make comprehensive and rigorous peer review more challenging than ever. Despite technological advancements that make possible remote connection, electronic documentation and sophisticated software to display plan information, unlike 20 yr ago, we can no longer afford to inspect each detail of every case we treat. The expanding volume of information to be reviewed, combined with the distinct nuances of a given plan, increase the chances of overlooking important elements of safety and effectiveness, especially if peer review is not performed by those whose specialty resides in the particular treatment site or technique being presented. Although the importance of peer review is indisputable, modern peer review is often rushed and sometimes approached as a mere fulfillment of legal and administrative requirements.

*Increase in workflow burden hinders comprehensive prospective peer review*. The rising complexity of treatment planning, the advent of new technologies, and a growing number of treatment options, have resulted in more extensive and time‐consuming documentation, alongside a plethora of regulations and additional quality checks that have increased the workload per plan. Altogether, these factors limit the time available to the review team for meaningful case discussion, making it increasingly difficult to adhere to recommendations for performing prospective peer review.^8^ This often results in postponing peer review until after treatment starts (retrospective review), although studies indicate that thorough prospective review identifies a larger number of issues with treatment plans.^29^ Reports suggest that participants are less likely to recommend changes to treatment plans once the patient has started treatment, likely due to an array of different cognitive biases and to avoid increasing workflow burden.^9^, ^12^, ^30^ Even when changes are recommended, they are more likely to be implemented if treatment has not yet started.^17^

*Upgrading modern peer review to meet the needs of modern radiotherapy*. Current peer review practices need to be upgraded to reflect high‐risk aspects of modern radiotherapy, and such changes need to be widely adopted. Peer review should not be viewed as one‐size‐fits‐all, but rather tailored to case complexity. Strategies to reduce the cognitive load of peer review, such as standardization in nomenclature,^31^ organization,^32^ and display of as many parameters as possible can and should be implemented. Institutional and interinstitutional guidelines should be developed and converted into treatment site‐specific care plans to maximize standardization of simulation‐to‐treatment processes (simulation, prescription, planning technique/goals, etc.) with the aid of evidence‐based recommendations, and knowledge‐based and artificial intelligence‐based tools.^33^, ^34^, ^35^, ^36^, ^37^ Noncompliant cases should be flagged for more careful review. When possible, automation tools and peer review platforms should be leveraged to expedite review without sacrificing quality. As emphasized above, the timing of peer review affects its effectiveness, making a case for plans to always be reviewed prior to treatment,^8^ including contour review before planning (i.e., contouring rounds).^12^

Finally, to truly achieve effective multidisciplinary peer review, a critical role needs to be attributed to radiation biologists to continually improve our understanding of the radiobiological consequences of different fractionation schedules, treatment modalities, retreatment doses, tissue reactions, etc. Evidence shows that integrating tissue‐specific radiobiological parameters into modern treatment plans for NSCLC can be informative on the effectiveness of the radiotherapy techniques being used.^38^ Understanding and foreseeing systemic reactions to radiotherapy, such as “interleukin storms”^39^ and radiation‐induced “*in‐situ* vaccination”^40^ in the advent of immunotherapy,^41^ and incorporating genomic biomarkers and other novel radiobiological parameters^42^ in treatment planning,^43^ have the potential for elevating peer review to meet the complex challenges of modern radiotherapy. We believe that radiobiologists need to return to the peer review table, as was the case 20 yr ago, in a structured, systematic way, immediately useful to the clinic and not relegated only to basic science laboratories.

In summary, to achieve a modern peer review that is as effective as 20 yr ago, comprehensive and thorough prospective peer review at different steps of the process needs to become once again an indispensable part of modern radiation treatment planning, with appropriate simplification and intensification of the process when needed, and perhaps most importantly, implementation of the equal contribution of the three distinct disciplines of Radiation Oncology.

## 4 REBUTTAL

### 4.A Lakshmi Santanam, PhD; Abhishek Solanki, MD; Anis Ahmad, PhD (FOR)

We thank our esteemed colleagues for their thoughtful arguments that peer review is essential for the quality and safety of the patients undergoing complex radiotherapy and agreeing that sophisticated software makes modern peer review in Radiation Oncology more effective than 20 yr ago.

The modern peer review process involving plan evaluation in a feedback environment from a multidisciplinary team is an effective strategy for assuring plan quality and patient safety as recommended by professional organizations such as ASTRO, ACR, and RANZCR.^8^, ^10^, ^44^

Uniformity in delineation patterns among physicians has been achieved with the help of standardized contouring protocols. In a study by Mitchell et al,^45^ six radiation oncologists contoured the radiation plans for patients undergoing prostatectomy before and after, providing a contouring atlas. With the help of the evidence‐based contouring protocol, the variability in target volume outlining was reduced significantly. Stepwise contouring guidelines and an atlas was demonstrated by Goodman et al^46^ to delineate the CTV in the postoperative irradiation of pancreatic cancer patients. These guidelines help the physicians in determining the areas at risk and minimizing the dose to normal tissues.

Recently, some centers have started daily peer review meetings and published the results. Modern peer review is one of the most effective ways of dealing with routine but controversial patient‐specific radiation oncology decisions. With the help of modern peer review, up to a quarter of contours may change.^47^ It has helped in standardizing clinical practice patterns to develop uniform treatment planning guidelines.

Quoting from the recent editorial article in PRO, "We are convinced that this is an obvious opportunity for our field; it is time to cut bait and say (once again) that preplanning peer review should be our standard, period."^48^ Critical aspects like shorter chart rounds, having a multidisciplinary team, encouraging safety culture by empowering participants to ask questions, incorporating known errors in Chart Rounds to QA the effectiveness, encouraging remote review via video conferences, should be the norm in peer review.^48^, ^49^ Treatment planning systems that can perform biological dose evaluations and dose summations when prior treatment reviews are mandated will help physicians understand the complexity of normal tissue sparing vs adequate coverage while prescribing dose or reviewing plans. Encouraging vendors to design peer review automation tools, auto‐segmentation tools, auto planning, plan evaluation, and biological dose evaluation tools can make the process faster and more efficient.

We agree that the issue of peer review for providers in solo practice can be incredibly challenging to address. However, innovative web‐based approaches are in the process of being tested.^50^ According to Reddeman et al.,^5^ our collective vision should be toward specialty‐specific peer review and comprehensive multidisciplinary peer review (e.g., tumor boards) for all the patients. There are several ongoing initiatives, including through ASTRO and the American Brachytherapy Society, to develop programs to aid sites with staffing limitations to perform peer review.

Numerous studies performed on the peer review process demonstrate the feasibility of performing prospective chart rounds in a format that occurs multiple times a week.^13^, ^51^, ^52^

We agree that the increase in workflow burden hinders comprehensive prospective peer review. Change happens slowly in healthcare, and improvement needs hard work. However, positive quality improvement is possible with carefully designed implementation of the plan, a good case for change, vision, realistic timelines, feedback, buy‐in, ownership from stakeholders and leaders, and accountability.

### 4.B Laura Padilla, PhD; Erina Vlashi, PhD; Patrizia Guerrieri, MD (AGAINST)

We certainly agree with the opposition that peer review is a critical step in ensuring quality care in radiation oncology. We also agree that modern technological advances facilitate information access and remote viewing, increasing the feasibility of engaging more team members regardless of physical location, including in other institutions. Finally, we agree that these modern advances have the potential to make peer review more effective. However, we are arguing that although 20 yr ago peer review operated with resources that were much more limited compared to what exists today, the available resources were utilized much more effectively. In other words, we maintain our position that while modern technology offers unprecedented opportunities for performing superior peer review, the available technology is not presently being employed to its full extent for optimizing the process. Additionally, the majority of institutions have yet to adopt prospective peer review.

There are examples in the literature of prospective peer review being successfully implemented,^12^, ^14^, ^21^, ^28^, ^53^ as the opposition has indicated, but this is not the current standard in the field. Statistics presented in the literature within the last 10 yr indicate that less than 40% of treatment plans are reviewed prior to treatment start.^54^ Furthermore, in a recently published editorial in the Red Journal, the authors describe chart rounds with peer review as “a weekly meeting where treatment plans of patients who are in their first week of treatment are peer reviewed,” further reiterating that retrospective review is the current standard in radiation oncology.^48^ Although the 10% plan modification rate resulting from retrospective peer review highlights the importance of the activity in providing quality patient care even when done retrospectively, this rate more than doubles in publications where peer review is done earlier in the treatment process.^14^, ^21^, ^28^, ^53^ This emphasizes once again that prospective peer review is superior and suggests that peer review in its current form is not as effective. Even with the increased rate of problem detection in prospective peer review, it is difficult to discern its absolute effectiveness as the true number of problematic plans is unknown. However, progress has been made to determine the detection rate of the widely implemented retrospective peer review format. Talcott et al recently published the results from a prospective study designed to determine the error detection rate during peer review.^49^ The authors created 20 plans containing errors and randomly inserted these plans into their institution’s weekly chart rounds (plans presented within the first week of the patient’s treatment) for peer review over 9 weeks. The results were sobering, revealing that a staggering 45% of the problematic plans were *not* successfully identified. Despite not having the equivalent data from 20 yr ago for comparison, it is clear that current peer review practices are ineffective.

We are not disputing the point that improvements have been made to peer review over the past 20 yr — of course, modern technological advancements have naturally enhanced peer review. However, we stand by our position that the rate of improvement in peer review has not kept pace with the expansion of plan complexity and workflow burden for radiation oncology team members. There is a clear need for rethinking the design and format of this practice and for effectively utilizing technology to optimize the information that participants need to process to successfully assess the quality of a plan. In contrast to 20 yr ago, we currently have a wider range of treatment techniques and encounter an increasing number of patients that require retreatments and multimodality treatment regimens. Additionally, we try to consider an ever‐increasing amount of potentially relevant information, that is, imaging, genetic testing, available biomarkers etc., when tailoring a patient’s treatment plan. These factors, and others, inevitably impose a cognitive load on peer review participants that is simply too great to allow for thorough, thoughtful, and effective plan review, especially for an extended period of time. Although the error detection rate significantly drops after the first 30 min, peer review sessions routinely last an hour, on average.^49^ Exacerbating this problem is the lack of protected time for team members to dedicate to peer review; those who participate often experience multiple interruptions that hamper their attention. This is also echoed in the aforementioned editorial,^48^ which re‐emphasizes our initial statement that in its current, rushed state, peer review is often approached as a mere fulfillment of legal and administrative requirements.

While it is ultimately impossible to support or debunk the proposition with certainty due to lack of hard data on the effectiveness of plan error detection from peer review over the past 20 yr, it is indisputably clear that current peer review practices in our field, as a whole, have not been sufficiently updated to keep up with technological and medical progress. Furthermore, cultural and behavioral barriers to peer review that were present 20 yr ago still remain today and require a full commitment to a culture of safety to be overcome.^50^ We all agree that the tools to start addressing these shortcomings are available, and that there are a few institutions leading the way on more effective peer review designs. However, until this becomes the norm and not the exception, we need to accept the fact that peer review in radiation oncology is not more effective today than 20 yr ago. Unless the field recognizes and accepts this, progress toward a truly effective peer review design that takes advantage of the full potential of modern technology will continue to trail behind medical practice, and we will still find ourselves debating this same issue 20 yr from now.

## CONFLICT OF INTEREST

The authors declare no conflict of interest.
